# Type I Interferons: Key Players in Normal Skin and Select Cutaneous Malignancies

**DOI:** 10.1155/2014/847545

**Published:** 2014-01-05

**Authors:** Aimen Ismail, Nabiha Yusuf

**Affiliations:** ^1^School of Medicine, University of Alabama at Birmingham, Birmingham, AL 35294, USA; ^2^Department of Dermatology, University of Alabama at Birmingham, VH 566A, 1670 University Boulevard, Birmingham, AL 35294, USA

## Abstract

Interferons (IFNs) are a family of naturally existing glycoproteins known for their antiviral activity and their ability to influence the behavior of normal and transformed cell types. Type I Interferons include IFN-**α** and IFN-**β**. Currently, IFN-**α** has numerous approved antitumor applications, including malignant melanoma, in which IFN-**α** has been shown to increase relapse free survival. Moreover, IFN-**α** has been successfully used in the intralesional treatment of cutaneous squamous cell carcinoma (SCC) and basal cell carcinoma (BCC). In spite of these promising clinical results; however, there exists a paucity of knowledge on the precise anti-tumor action of IFN-**α**/**β** at the cellular and molecular levels in cutaneous malignancies such as SCC, BCC, and melanoma. This review summarizes current knowledge on the extent to which Type I IFN influences proliferation, apoptosis, angiogenesis, and immune function in normal skin, cutaneous SCC, BCC, and melanoma.

## 1. Introduction

Interferons (IFNs) are a group of naturally existing glycoproteins that are secreted by cells in response to viral infections as well as synthetic and biologic inducers. Since the discovery of IFNs more than 50 years ago, *in vitro* and *in vivo* assays have demonstrated a diverse spectrum of biological activity, including antiviral, antiproliferative, and immunomodulatory properties [1]. Type I interferons include IFN-*α*, IFN-*β*, IFN-*ε*, IFN-*κ*, and IFN-*ω*. Type II interferons include IFN-*γ*, and type III interferons include IFN-*λ* [1–3].

Type I interferons (IFN-*α*, IFN-*β*) bind to cell surface receptors with two distinct subunits: IFN-*α* receptor 1 and IFN-*α* receptor 2. This binding triggers phosphorylation of janus kinase 1 (JAK1) and tyrosine kinase 2 (TK2), members of the Janus kinase family of receptor-associated tyrosine kinases. These kinases proceed to phosphorylate signal transducers and activators of transcriptions 1 and 2 (STAT1 and STAT2), which belong to a group of latent cytoplasmic transcription factors. The activated STAT1 and STAT2 proteins complex with p48 protein to form the IFN-stimulated gene factor 3 (ISGF3) transcription factor. ISGF3 translocates to the nucleus, where it binds to IFN-stimulated response elements in the promoters of type I IFN-responsive genes and thereby activates transcription [4, 5].

IFN-*γ* signals through the cell surface receptor IFNGR, which consists of IFNGR1 and IFNGR2 chains, impacting distinct but related pathways to those of type I IFN. IFN-*λ* signals through the unique receptors IFNLR1 and IFN-10R2 [3].

Among the interferons, IFN-*α*2 has been the most broadly evaluated clinically, and its three commercially available subspecies include IFN-*α*2a, IFN-*α*2b, and IFN-*α*2c [3]. With the approval of IFN-*α*2a and IFN-*α*2b for the treatment of hairy cell Leukemia in 1986, IFN became the first recombinant cytokine to be licensed in the United States for the treatment of a malignancy. Since then, other approved antitumor applications for IFN-*α*2a or IFN-*α*2b include AIDS-related Kaposi's sarcoma, chronic myelogenous leukemia, follicular lymphoma, and malignant melanoma [6]. Currently, the only approved agents for the adjuvant treatment of resected melanoma that is at high risk of recurrence are IFN-*α*2b in Europe and the United States, pegylated IFN-*α*2b in the United States and Switzerland, and IFN-*α*2a in Europe. High-dose IFN-*α*2b (HDI) is the approved dosing regimen in the United States for American Joint Committee on Cancer (AJCC) stage IIB-III melanoma and consists of an induction phase of 20 MIU/m^2^ intravenously (IV) 5 times/week for 4 weeks followed by a maintenance phase of 10 MIU/m^2^ subcutaneously (SC) 3 times/week for 48 weeks [7].

The results of a metaanalysis of 18 randomized controlled trials published between 1995 and 2011 demonstrate that adjuvant IFN-*α* significantly increases both disease-free survival and, to a lesser extent, overall survival in high-risk (AJCC TNM stage II-III) cutaneous melanoma [8]. Moreover, numerous studies have demonstrated the efficacy of intralesional IFN-*α*2a and IFN-*α*2b for the treatment of cutaneous squamous cell carcinoma (SCC) and basal cell carcinoma (BCC) [1, 9–16]. However, there exists a dearth of knowledge on the precise antitumor action of IFN-*α*/*β* at the cellular and molecular levels in cutaneous malignancies such as SCC, BCC, and melanoma. This review serves to summarize current knowledge on the extent to which type I IFN influences proliferation, apoptosis, angiogenesis, and immune function in SCC, BCC, and melanoma. Considerably more is known regarding the mechanism of IFN action in melanoma than in SCC and BCC, and this discrepancy is reflected in the content of this review.

## 2. Effect on Normal Keratinocytes and Melanocytes

### 2.1. Antiproliferative Effects

Various studies have shown type I IFN to have an antiproliferative, prodifferentiation effect on normal keratinocytes and melanocytes. Experiments by Yaar et al. showed that cultures of human keratinocytes supplemented with 2500 units/mL of either IFN-*α* or IFN-*β* demonstrated a mean growth inhibition of 70% at 7 days compared with control cultures. Moreover, IFN-*α* and -*β* promoted keratinocyte terminal differentiation as demonstrated by increased cornified envelope formation and cell shedding in IFN-treated cultures compared to controls. The effects of IFN-*α* and -*β* on growth and terminal differentiation were reversible upon withdrawal of IFN from the medium [17].

Similarly, Nickoloff et al. showed that incubation of cultured human keratinocytes with 19.8 × 10^3^ units/mL IFN-*α* resulted in an approximately 30% decrease in number of attached keratinocytes at day 8 compared to control [18].

Bielenberg et al. showed that, in tissue samples of normal murine and human skin, keratinocytes in the basal layer did not express IFN-*β*, whereas those in the suprabasal layers did, and this expression of IFN-*β* directly correlated with production of differentiation markers. The *in vitro* expression of IFN-*β* by undifferentiated, growth-arrested murine keratinocytes suggested that the production of IFN-*β* by terminally differentiated cells was associated with cessation of proliferation. Further, tissue samples from neither human nor transgenic mouse squamous cell carcinomas expressed significant levels of IFN-*β* [19].

Krasagakis et al. demonstrated a strong growth inhibition of normal human melanocytes by IFN-*β* in a dose- and time-dependant manner in 6- and 12-day assays in both RMM and CMM media (at 12 days, 10,000 IU/mL IFN-*β* led to 80% growth inhibition in CMM and 77% growth inhibition in RMM compared to controls). In contrast 10,000 IU/mL of IFN-*α* showed no effect on melanocyte proliferation in RMM but did lead to a 24% growth inhibition in CMM compared to controls in 12 days. CMM is TPA- and serum-free complete melanocyte medium, and RMM is its mitogen-reduced variant [20].

### 2.2. Antiangiogenesis Effects

Mouse IFN-*α*/*β* have been shown to inhibit experimental wound healing in mice through the inhibition of proliferation of many different cell types, including endothelial, epidermal, and connective tissue cells [21].

McCarty et al. implanted gelfoam sponges in IFN-*α*/*β* receptor −/− mice and IFN-*α*/*β* receptor +/+ mice and proceeded to induce endothelial cell migration and proliferation with 200 ng/mL of the proangiogenic factors bFGF, VEGF, and TGF-*α*. Sponges that were recovered from IFN-*α*/*β* R −/− mice demonstrated a significantly higher number of blood vessels than did those recovered from IFN-*α*/*β* R +/+ mice, indicating that IFN sensitivity of surrounding tissue was necessary for inhibition of angiogenesis around the sponges [22].

### 2.3. Immunomodulatory Effects

In experiments by Niederwieser et al., cultured human keratinocytes exposed for 72 hours to 500 units/mL IFN-*α* showed 63% class I MHC antigen expression, compared to 70% for IFN-*γ* at 500 units/mL, and 51% for untreated keratinocytes. In their experiments, induction of class II MHC antigen expression was a feature of IFN-*γ*-, and not IFN-*α*-, treated cultures [23].

Krasagakis et al. showed that 95–100% of normal cultured human melanocytes grown in melanocyte growth medium (MGM) expressed HLA class I antigens, but none of them expressed HLA-DR, a class II antigen. Treatment with 1000 IU/mL of IFN-*α* or -*β* resulted in a stronger expression of HLA class I antigens, with IFN-*β* having a greater effect than IFN-*α*. Moreover, while IFN-*α* induced no change in HLA-DR expression by normal human melanocytes, IFN-*β* induced de novo expression of HLA-DR in ≤20% of the cultured cells. Interestingly, IFN-*γ* had the greatest effect on induction of HLA-DR, with 95% of melanocytes HLA-DR-positive at 1000 IU/mL IFN-*γ* [20]. The effects of type I IFNs on keratinocytes and melanocytes have been summarized in Table 1.

## 3. Cutaneous Squamous Cell Carcinoma

### 3.1. Antiproliferative Effects

The growth inhibitory and cell cycle effects of IFNs-*α*,*β* have been evaluated in numerous human skin SCC cell lines. In SCL-1 cells, Nickoloff et al. showed that recombinant IFN-*α* at 1.98 × 10^2^ U/mL resulted in 89% of control in cell number on day 5, compared to 78% at 2.18 × 10^2^ U/mL of recombinant IFN-*β* [24]. Naito et al. reported that cell number of A431 human squamous cell carcinoma cells markedly decreased with IFN-*β* at 5000 U/mL [25].

IFN-sensitive SRB12-p9 cells were more sensitive to the growth inhibitory effect of treatment with 100 U/mL IFN-*α* continuously for five days than were IFN-resistant SRB1-m7 cells (53.8% and 19.1% growth inhibition compared to controls, resp.). In SRB12-p9 cells, 100 U/mL IFN-*α* induced a partial G1/0 arrest (57.4% of cells in G1/0) compared to controls (48.6% of cells in G1/0), whereas this effect was not seen in SRB1-m7 cells [26].

Yaar et al. showed that growth of the human epidermal squamous cell carcinoma cell line SCC-12B.2 is inhibited to a lesser degree by IFN-*α* and IFN-*β* than normal human keratinocytes *in vitro*; this result indicates that loss of sensitivity to IFN is a characteristic of malignant cells [17].

### 3.2. Proapoptotic Effects

IFN-*α* produced a greater than twofold increase in apoptosis (4.4% apoptosed cells) compared to controls (1.9% apoptosed cells) in SRB12-p9 cells, whereas no increase in apoptosis was seen with IFN-*α* treatment in IFN-resistant SRB1-m7 cells [26].

Rodriguez-Villanueva and McDonnell reported that, beginning 48 hrs after addition of 100 IU/mL IFN-*α*-2b, SRB-12 cells exhibited ultrastructural alterations associated with apoptotic cell death on transmission electron microscopy as well as DNA “laddering” on agarose gel electrophoresis. Overexpression of bcl-2 only partially blocked the direct cytotoxic effects of IFN-*α* in SRB-12. In contrast, the SRB-1 cell line showed no significant cytotoxicity to exogenous IFN-*α*, even in the presence of concentrations up to 10^5^ IU/mL [27].

### 3.3. Immunomodulatory Effects

IFN-*β* at doses of 50, 500, and 5000 U/mL failed to induce HLA-DR antigen expression in A431 human squamous cell carcinoma cells after a 24 hr incubation, whereas a similar treatment with IFN-*γ* significantly enhanced HLA-DR expression in a dose-dependant manner. Moreover, 24 hr incubation of IFN-*β* at 50 or 500 U/mL with 5 U/mL IFN-*γ* was found to decrease the IFN-*γ*-induced expression of HLA-DR antigens and mRNA in a dose-dependant manner. It was similarly found that IFN-*α* at 500 U/mL reduced IFN-*γ*-induced HLA-DR expression in A431 cells [25].

### 3.4. Miscellaneous Findings

In an immunohistochemical study by Clifford et al. involving 16 surgical specimens of aggressive human skin SCCs, pairwise comparisons for STAT1*α*/*β*, STAT2, p48, and STAT3*α*/3*β* revealed significantly lower staining intensity for some or all of these proteins in tumor cells compared with adjacent nonmalignant epidermal tissue. These results indicate that a decrease in IFN responsiveness may lead to tumorigenicity [4]. A follow-up immunohistochemical study by the same group revealed a significant decrease in expression of one or more ISGF-3 proteins in 19 of 25 patients with actinic keratosis compared to matched normal skin. This result indicates that a decrease in responsiveness to endogenous IFN likely represents an early event in skin carcinogenesis [5]. Since STAT2 is thought to be the only STAT specific for the IFN-*α* pathway, Clifford et al. also conducted experiments to permanently block IFN-*α* signaling in a skin cell-based system through the forced expression of double negative STAT2 protein. In experiments involving the IFN-*α* sensitive SRB12-p9 human skin SCC cell line, dnSTAT2-expressing clones treated for four days with 100 IU/mL IFN-*α* showed 15% growth inhibition compared with 47.5% for parental SRB12-p9 cells. Moreover, dnSTAT2 expression suppressed the upregulation of several IFN-*α*-inducible genes identified by cDNA microarray screening. These findings led the group to conclude that the cell-growth inhibitory effect of IFN-*α* in skin cells requires an intact STAT2 protein and is therefore mediated by the ISGF-3 complex [28]. The effects of type I IFNs on squamous cell carcinoma have been summarized in Table 2.

## 4. Basal Cell Carcinoma

### 4.1. Antiproliferative Effects

One group used real-time PCR to analyze opioid growth factor receptor expression in four primary basal cell carcinoma-derived cell lines treated with imiquimod or IFN-*α* for 24 hr. IFN-*α* upregulated opioid growth factor receptor expression in 2 of the 4 cell lines, whereas imiquimod did not induce a chance in opioid growth factor receptor expression in any of the cell lines [29]. Opioid growth factor is known to act as a negative regular of cell proliferation through DNA synthesis pathways [30], so this finding may represent a novel growth-inhibitory mechanism of action for IFN-*α*.

### 4.2. Proapoptotic Effects

In a study of 15 patients with histologically proven nodular BCC, 9 of whom were treated with intralesional IFN-*α*-2b, the BCC cells of untreated patients constitutively expressed CD95L, whereas the BCC cells of treated patients not only expressed CD95L but also became CD95 positive. This concomitant expression of CD95L and CD95 eventually led to cell death by suicide and fratricide, with the majority of apoptotic cells in the center, rather than the periphery, of BCC nests. The IFN-*α*-induced CD95 expression in BCCs was either a direct effect of the drug or indirectly mediated through cytokines produced by the CD4^+^ T cell predominant peritumoral lymphoid infiltrate [31].

### 4.3. Immunomodulatory Effects

IL-10 is potent immunosuppressive cytokine, and previous studies have discovered elevated levels of IL-10 mRNA in BCC and SCC compared to matched PBMCs and seborrheic keratoses, respectively. These studies have revealed that neutralization of tumor-produced IL-10 by monoclonal antibodies can restore anti-tumor T-cell recognition [32, 33]. Kim et al. found a decrease in IL-10 mRNA levels in excisional biopsy specimens from four BCCs after IFN-*α* treatment compared to pretreatment levels as well as a decrease in IL-10 mRNA levels in 2 BCC-derived cell lines and 2 SCC-derived cell lines following 24-hr IFN-*α* treatment. In these experiments, treatment of BCCs with IFN-*α* was associated with reduction in malignant cells on histologic examination [32].

Buechner treated four patients with nodular basal cell carcinomas with intralesional injections of IFN-*α*-2b (1.5 million IU per injection) three times a week for two weeks. Four weeks after completion of therapy, histopathologic examination of biopsy specimens revealed resolution of BCC and a dense dermal mononuclear cell infiltrate. Immunohistochemical analysis revealed the dermal infiltrate to contain CD4^+^ and CD8^+^ T cells in ratios ranging from 2 : 1 to 3 : 1, CD22 cells (B cells), IL-2 receptor-expressing cells, and NK cells. CD1+ cells (Langerhans cells) were observed in the epidermis, dermoepidermal junction, and around and within dermal BCC nodules. Most of the dermal infiltrate stained for HLA-DR, although tumor cells did not; there was focal expression of HLA-DR on keratinocytes, particularly in areas of dense inflammatory infiltrate. A considerable number of HLA-DR^+^ dendritic cells and Langerhans cells were present at the periphery of tumor masses, in close proximity to HLA-DR^+^-activated T cells [34].

In an analogous study by Mozzanica et al. six patients with nodular (2) or superficial (4) basal cell carcinomas were treated with intralesional injections of IFN-*α*-2b (1.5 million IU per injection) three times a week for three weeks. Immunohistologic study was done before the start of IFN therapy and after two weeks of therapy. In analysis of peritumoral infiltrate, treatment with IFN-*α* led to an increased proportion of CD3^+^ cells (53% versus 66.5%), with an increase in the CD4/CD8 ratio from 1.4 to 1.9. In analysis of intratumoral infiltrate, treatment with IFN-*α* led to an increased proportion of CD3^+^ cells (8.0% versus 13.5%), with an increase in the CD4/CD8 ratio from 1.5 to 3.2. In both peritumoral and intratumoral infiltrates, the pre- and posttreatment changes in percentage of cells that expressed HLA-DR, CD1 (Langerhans), CD14b (monocytes/macrophages), CD56 (natural killer), CD20 (B cells), and CD15 (granulocytes) were not significant. 8 weeks after completion of therapy, 2 BCCs were cured and 4 showed clinical and histologic signs of improvement [35]. The effects of type I IFNs on basal cell carcinoma have been summarized in Table 3.

## 5. Melanoma

### 5.1. Antiproliferative Effects

Dose response curves produced by Johns et al. showed the following order of potency of inhibition for the cell lines SK-MEL-28, Hs294T, HT144, and SK-MEL-3: IFN-*β* > IFN-*α*-2b >IFN-*α*-4a [36]. Krasagakis et al. showed that 10,000 IU/mL of IFN-*β* and -*α* inhibited the proliferation of SKMel-28 melanoma cells at 5 days by 78% and 59% of the controls, respectively [20]. For the cell lines LiBr and SK-MEL-1, the order of inhibitory potency was IFN-*β* > IFN-*α*-2b = IFN-*α*-4a. The greater antiproliferative potency of IFN-*β* compared to IFN-*α*-2a was also borne out in experiments using xenografts of the melanoma cell line LiBr in nude mice. In competitive binding assays in HT144, SK-MEL-28, MM418, and MM96 cell lines, the order of competition for the IFN receptor was the same as that for antiproliferative potency, IFN-*β*> IFN-*α*-2b > IFN-*α*-4a [36].

Dose-dependent inhibition of proliferation of SK-MEL-2 and SK-MEL-24 cells was seen after treatment with IFN-*α*-2b or IFN-*β*-1a, with greater inhibition by IFN-*β*-1a. Treatment with IFN-*α*-2b and IFN-*β*-1a also resulted in decreased proliferation index of human melanoma xenograft tumors as manifest by immunohistochemical staining with Ki-67; again, IFN-*β*-1a-treated tumors showed less staining with Ki-67 than did IFN-*α*-2b-treated tumors [37]. In experiments by Garbe et al., IFN-*α* and IFN-*β* both showed concentration-dependant inhibition of proliferation of three melanoma cell lines, but IFN-*β* had the smallest IC_50_ for all three cell lines tested. Surprisingly, IFN-*α* and IFN-*β* decreased the proportion of terminally differentiated melanoma cells to 56–97% of untreated cultures [38]. Numerous other experiments have likewise demonstrated the dose-dependant antiproliferative effects of interferons as well as the greater effect of IFN-*β* compared to IFN-*α*, on melanoma [39–45].

As a final example, in four cell lines derived from human melanoma metastases (JKM86-4, 5, 8, and 9), IFN-*β* at 50–5000 U/mL had a stronger inhibitory effect than the same concentration of IFN-*α* in all cell lines. The genes for IFN-*α* and IFN-*β* are localized to chromosome 9p, and the antiproliferative effect of IFN-*α* and -*β* was more pronounced in the two cell lines that expressed the highest levels of 9p per cell (4 copies and 2.8 copies in JKM86-5 and JKM86-8, resp.), indicating that cell lines with more copies of 9p are more sensitive to IFN. IFN receptor genes are located on 21q, and copies of this chromosome did not appear to influence interferon sensitivity in the four cell lines [46].

### 5.2. Proapoptotic Effects

Seventy-two-hour Annexin V apoptosis assays as well as ninety-six-hour TUNEL apoptosis assays involving three melanoma cell lines showed an increasing induction of apoptosis at higher doses of IFN-*β*; no increase in apoptosis occurred with IFN-*α*2, even at the highest dose (1000 units/mL), in any of the three cell lines [44]. In human melanoma IGR 1 cells, the apoptosis-promoting effect of IFN-*β* at 500 IU/mL was time-dependant and greater than that of IFN-*α* at all time points [39]. Kubo et al. showed that IFN-*β* induced apoptosis dose-dependently in 7 melanoma cell lines as well as induced cleavage of caspase 3 in these cell lines [42]. The number of apoptotic cells in human melanoma xenograft tumors was significantly increased in IFN-*α*-2b- and IFN-*β*-1a-treated tumors compared with untreated tumors, with IFN-*β*-1a having a greater apoptotic effect than IFN-*α*-2b [37].

Cyt c was undetectable in the cytosolic fraction of untreated WM9 cells but increased in a time-dependant manner with IFN-*β*, but not IFN-*α*2, treatment. This phenomenon in WM9 cells was coupled with increased activity of caspases 3, 8, and 9. Lastly, IFN-*β* induced TRAIL mRNA expression in apoptosis-sensitive melanoma cell lines tested, whereas IFN-*α*2 did not. Together, these findings led Chawla-Sarkar et al. to conclude that IFN-*β* induces apoptosis through the production and secretion of TRAIL protein, which acts in an autocrine or paracrine manner to activate its death receptors on neighboring melanoma cells.

Regardless of their sensitivity to either cytokine alone, melanoma cell lines treated with IFN-*β* for 16–24 hrs before addition of TRAIL showed apoptosis of >30% of cells. Three such cell lines demonstrated cleavage of XIAP following combination treatment, whereas resistant cell lines did not. XIAP normally inhibits caspases 3 and 9 and has been shown to be cleaved in TRAIL-treated cells. IFN-*β* may sensitize cells to TRAIL through induction of XAF-1, which is a negative regulator of XIAP [47].

### 5.3. Antiangiogenesis Effects

Representative interferon-stimulated gene products were quantified in the serum of 10 patients with cutaneous metastatic melanoma after one month of daily injections with IFN-*β*1a at a dose of 12 × 10^6^ IU/m^2^ on days 1–14 and 18 × 10^6^ IU/m^2^ on days 15–29. The results showed significant increases in TRAIL, IL-1RA, CCL2, CCL8 (anti-angiogenic), CXCL10 (anti-angiogenic), CCL20, and CXCL8. There was a moderate decrease in the proangiogenic VEGF-A and CXCL5. In this study, IFN-*β*1a at a maximally tolerated dose led to tumor regression in only 1 out of 17 patients with cutaneous metastatic melanoma [48].

In a study involving 9 human melanoma cell lines, treatment of cells with 2000 U/mL IFN-*α* decreased VEGF secretion by 40–60% in VEGF-high cell lines, but not in VEGF-low cell lines [49]. Protein levels of VEGF-C and VEGFR-3 in SK-MEL-24 cells decreased in response to *in vitro* treatment with IFN-*α*2b or IFN-*β*1a, with IFN-*α*2b showing an earlier and more sustained response compared with IFN-*β*1a. Moreover, treatment with IFN-*α*2b or IFN-*β*1a also decreased secretory VEGF-C levels, with a superior effect by IFN-*α*2b [37]. In human melanoma IGR 1 cells, treatment with 500 IU/mL of either IFN-*α* or IFN-*β* significantly and similarly led to a decrease in VEGF production compared to controls [39].

Decreased levels of VEGF-C and VEGFR-3 were also seen in human melanoma xenograft tumors following IFN-*α*2b or IFN-*β*1a treatment. In human melanoma xenograft tumors, microvessel density was decreased by comparable amounts in tumors treated with IFN-*α*2b or IFN-*β*1a compared with the control. However, lymphatic vessel density was significantly decreased in xenograft tumors treated with IFN-*α*2b compared with either IFN-*β*1a-treated tumors or controls [37].

### 5.4. Immunomodulatory Effects

Studies have suggested that the effectiveness of type I interferon against melanoma is owed largely to indirect, immunomodulatory antitumor effects. In an immunocytochemical study involving fine needle aspirates from 21 patients with systemic metastatic malignant melanoma studied before initiation of IFN-*α* treatment, 10 out of 11 patients with moderate to high numbers of infiltrating CD4^+^ lymphocytes achieved tumor regression, while 9 out of 10 patients with low numbers of these cells had progressive disease. Similar results were found in 20 patients with regional metastatic disease. This importance of the presence of infiltrating CD4^+^ lymphocytes for the therapeutic effect of IFN-*α* shows that one important anti-tumor effect of IFN-*α* is to enhance immune reactivity toward the tumor [50].

Another study measured the recruitment of CD4^+^ cells close to tumor cells in resected metastases following treatment with IFN-*α* for 0–3 weeks in 26 IFN-treated and 10 untreated patients with regional metastatic melanoma. IFN-*α* treatment resulted in moderate to high numbers of CD4^+^ cells infiltrating close to tumor cells in 12 out of 26 metastases compared to 1 out of 10 metastases from untreated patients [51].

Moschos et al. conducted immunohistochemical analyses on biopsy specimens from 20 patients with stage IIIB-C melanoma before and after high-dose IFN-*α*-2b. Clinical responders showed a significantly greater increase in the number of tumor-infiltrating CD11c^+^ and CD3^+^ cells. HDI did not, however, appear to affect peritumoral infiltrates, or to alter angiogenesis, HLA expression, proliferation, or apoptosis [52].

In the first report on the testing of IFN-*α* as an adjuvant in the vaccination of cancer patients, 7 stage IV melanoma patients were injected with MART-1 and gp-100 peptides, and IFN-*α* was administered in close spatial and temporal proximity to the peptide vaccine. 3 of the 7 patients showed disease stabilization following vaccination. PBMC studies showed a significant increase in peptide- and melanoma-specific CD8^+^ T lymphocytes in 5 patients, increase in antigen-specific effector-memory (CD45RA^−^CCR7^−^) cells in the 3 patients with stable disease, and increase in percentage of CD14^+^CD16^+^ monocytes in all 7 patients. There was enhanced antigen-presenting cell function and IP-10/CXCL10 production by postvaccination monocytes in the patients with stable disease [53].

In a study involving a cohort of 21 patients with stage II or III melanoma treated with low-dose IFN-*α* for more than 12 months, blood samples were obtained before treatment, and at 3, 6, 9, and 12 months after initiation of treatment. During this time, there was a steady decrease in the number of peripheral blood circulating total CD3^+^ T lymphocytes as well as a decrease in the CD3^+^CD4^+^ and CD3^+^CD8^+^ lymphocyte subsets. The level of CD3-CD56^+^ NK lymphocytes was significantly decreased by 1 year. At one year there was a significant decrease in myeloid as well as plasmacytoid dendritic cells, with a more marked depletion of the latter dendritic cell subgroup. Total blood circulating monocytes (CD14^+^) were not significantly decreased but did show an increased fluorescence intensity of MHC class I molecules. IP-10/CXCL10 levels also increased during the treatment period [54].

Tsavaris et al. treated 14 melanoma patients with local recurrence or distant metastases with IFN-*α*-2b subcutaneously 3 times per week; the dose was increased from 5 × 10^6^ IU/day for the first week to 10 × 10^6^ IU/day for the second week and to 15 × 10^6^ IU/day thereafter. Two months after therapy with IFN-*α*-2b, 5 patients showed partial response and 9 exhibited progressive disease. *In vitro* studies of T cell function and cytokine production showed that, during therapy with IFN-*α*-2b, deficient immune responses were restored to almost normal levels in responders, whereas no significant improvement was seen in patients who had progressive disease. Specifically, responders showed a more than 25–40% increase in proliferation in autoMLR and alloMLR (MLR, mixed lymphocyte reaction), IL-2 production by T cells, IL-2 sensitivity of T cells, and IL-1 production by monocytes [55].

The effect of IFN-*β* on tumor infiltration by immune cells has likewise been investigated. Excisional biopsy specimens from metastatic skin lesions that were injected with IFN-*β* once weekly for four weeks showed increased tumor infiltration by TIA^+^, CD8^+^, and CD4^+^ cells. Additionally, there were significant numbers of infiltrating HLA-DR^+^ cells within the metastatic tumors that had received IFN-*β* injections compared with noninjected tumors (56.3% versus 10.4%). There was no significant difference in dendritic cell infiltration with and without IFN-*β* treatment [56]. After local injection of IFN-*β* (10^6^ U/injection 5 times over 5 successive days) into B16-F10 melanoma tumors in C57BL/6 mice, analysis of interstitial infiltrate showed 21–50% T cells and <5% NK cells; similarly, tumor nest infiltrate contained 5–20% T cells compared to 0% NK cells, indicating that the immune response was primarily T-cell-mediated [57].

IFN-*α* also appears to directly or indirectly modulate the expression of TNF-*α* and IL-8 in tumor cells. Melanoma metastases from 37 patients were stained for TNF-*α*: 16 metastases were from untreated patients and 21 were from patients treated with IFN-*α*. Significantly more metastases from IFN-*α*-treated patients had a low TNF-*α* staining score compared with metastases from untreated patients. Moreover, a low TNF-*α* staining score correlated with histopathologic regression of tumors [58]. In contrast, in a study that examined serum cytokine levels in IFN-*α*-treated patients, baseline levels of TNF-*α* for patients showing relapse under therapy were significantly lower than baseline levels of TNF-*α* for patients without relapse [59].

Although IFN-*α* and IFN-*β* alone did not inhibit steady state IL-8 production in three metastatic melanoma variants, they did inhibit IL-1*β* or TNF-*α*-mediated upregulation of IL-8 mRNA, with a more potent effect by IFN-*β* compared to IFN-*α*. These findings are notable since IL-8 is an autocrine growth factor for human melanoma cells and directly correlates with their metastatic potential [60].

Peripheral blood lymphocytes from three healthy donors were incubated with each of three irradiated primary melanoma cultures. 1000 U/mL IFN-*α* was added to the co-cultures at days 0, 3, 6, and 9, and on day 10 the ability of PBL to lyse radiolabeled melanoma cells was measured. IFN-*α* was a potent stimulator of anti-melanoma lytic activity. When the NK cell target K562 was added to the killing assay to inhibit NK cell-mediated lysis, a considerable fraction of the IFN-*α* cytolytic activity remained, demonstrating that IFN-*α* stimulated both NK and CTL generation. To show that essentially all the lytic activity observed in the presence of K562 cells was due to a T cell receptor-mediated mechanism, they used a combination of anti-CD3 and anti-CD8 antibodies to block the activity. MHC class I, but not class II, expression was upregulated by IFN-*α* in two of the primary melanoma cultures, and this represents a possible mechanism by which IFN-*α* can stimulate CTL generation [61]. In a study assessing NK cell activity against three melanoma cell lines, IFN-*β* and IFN-*α*2 showed a similar, dose-dependant augmentation of NK cell-mediated cytotoxity, and this augmented NK cytotoxicity did not correlate with antiproliferative effects of the IFNs [43].

In addition to many of the studies above, numerous others have demonstrated a stimulatory effect of type I interferons on MHC classes I and II expression on melanoma and immune cells. Treatment of murine B16 melanoma cells with IFN-*α*, IFN-*α*/*β* and IFN-*γ* resulted in enhanced class I H2 antigen expression, with IFN-*γ* having the greatest effect [62]. An increase in *β*_2_ microglobulin cell surface expression with IFN-*α* treatment (500 units/mL for 72 hrs) was observed in Hs294T and Hs695-L melanoma cell lines [63]. In a study that involved administering escalating doses of IFN-*α* to 9 melanoma patients for three weeks, isolation of PBMCs at regular intervals revealed elevated class I MHC at mRNA, translational, and plasma membrane levels [64]. In a study of 25 patient-derived melanoma cell lines, all cell lines expressed class I MHC antigen, and all three interferons (IFNs-*α*, *β*, *γ*) significantly enhanced mRNA levels, protein synthesis, and membrane expression. In the 22 cell lines displaying baseline expression of HLA class II antigen, IFN-*γ* increased the levels of class II mRNA, protein synthesis, and surface expression, whereas a significant upregulation of class II transcripts and protein levels by IFN-*α* or IFN-*β* was found in only two cell lines [65]. Experiments in the human melanoma cell line MeWo and its metastatic variant MeM 50-10 demonstrated increasing expression of HLA class I antigen with IFN-*α* (2000 units/mL), IFN-*β* (3000 units/mL), and IFN-*γ* (1000 units/mL) treatment, respectively. Induction of MHC Class II antigen was seen in MeM 50-10 cells only, and even then only with IFN-*β* (3000 units/mL) or IFN-*γ* (1000 units/mL), with a greater enhancement by the latter [66].

IFN-*β*, but not IFN-*α*, was shown to increase mRNA and protein expression of melanocytic tumor-associated antigens (Melan-A/MART-1, gp100, MAGE-A1) in 15 melanoma cell lines, inducing susceptibility to lysis by cytotoxic T lymphocytes [67].

In the presence of IFN-*α* and granulocyte/macrophage colony-stimulating factor (GM-CSF), monocytes differentiate into dendritic cells termed IFN-DCs. These IFN-DCs are effective in taking up antigens, migrating to lymph nodes, producing T-helper 1 mediators, and stimulating T- and B-cell responses. IFN-DCs may therefore be promising adjuvants for cancer immunotherapy targeting melanoma [68].

### 5.5. Miscellaneous Findings

Experiments with human malignant melanoma tissues and cell lines have shown that proinflammatory cytokines, such as IL-1*α*/*β* and TNF-*α*, produced by melanoma cells activate p38 kinase to promote the IFN-*α*/*β*-independent pathway of IFNAR1 degradation. By linking tissue inflammation with decreased cell sensitivity to the effects of type I IFN, these findings help to explain the decreased sensitivity of melanoma to the antitumorigenic effects of endogenous as well as therapeutically administered exogenous IFN-*α*/*β* [69].

Another group found that the pSTAT1/pSTAT3 ratio in tumor cells at baseline may serve as a useful prognostic predictor in cutaneous melanoma and a predictor of therapeutic effect for IFN-*α*2b. STAT1 restricts cell growth and mediates the antitumor effects of IFN-*α*, while STAT3 is associated with melanoma tumor progression and host immunosuppression. Tissue samples from stage IIIB patients were obtained before and after 20 doses of HDI therapy. Higher pretreatment pSTAT1/pSTAT3 ratios in tumor cells were associated with longer overall survival, and pSTAT1/pSTAT3 ratios were augmented by HDI in melanoma cells as well as in lymphocytes. The group concluded that downregulation of STAT3 and pSTAT3 by HDI in melanoma and host immune cells is central to the immunomodulatory effect of IFN-*α* [70].

In *in vivo* prospective studies involving patients with a clinical history of resected primary melanoma who had at least four atypical nevi, systemic low dose IFN-*α* treatment for three months led to decreased detection of Stat3/Stat3 and Stat1/Stat1 homodimers and Stat1/Stat3 heterodimer in atypical nevi excised after completion of treatment compared to those resected before IFN-*α* treatment. Moreover, IFN-*α* treatment led to dephosphorylation of constitutively activated Stat3 protein in atypical nevi [71].

Simons et al. suggested a way to select patients for high-dose interferon therapy based on their peripheral blood lymphocyte (PBL) IFN signaling patterns. They measured IFN signaling responses in PBL from 14 stage IIIB-C melanoma patients taken at baseline and at day 29 of neoadjuvant HDI therapy. The induction of pSTAT1 from IFN-*α* stimulation was assessed by phosflow in PBMCs. Those patients with good clinical outcome over the 4-wk induction phase had a significant increase in STAT1 activation in peripheral blood T cells upon IFN-*α* stimulation from day 0 to day 29. Responding patients showed a lower IFN-*α*-induced pSTAT1 response at day 0 compared to nonresponding patients [72].

Most clinical studies of the role of type I interferon in melanoma have focused on IFN-*α* rather than IFN-*β*. In Japan; however, natural IFN-*β* is approved and widely used as adjuvant therapy for melanoma. In a Japanese study of 46 patients with stage II and III primary cutaneous melanoma, 21 patients were treated with low-dose IFN-*β* maintenance therapy, and 25 patients underwent observation alone. Overall survival (OS) and relapse-free survival (RFS) were significantly worse in the observation group: mean OS was 56.3 months for the observation group and 90.6 months for the IFN group, and mean RFS was 54.9 months for the observation group versus 90.3 months for the IFN group [73]. The effects of type I IFNs on melanoma have been summarized in Table 4.

## 6. Conclusion

In summary, the precise mechanism by which type I interferons exert their antitumor effects in SCC, BCC, and melanoma is the subject of ongoing study, and much remains to be elucidated. Although surgical excision remains the preferred mode of treatment for BCC and SCC, intralesional IFN-*α*/*β* is a reasonable alternative to surgery for patients with poor hemostasis, those at high risk for poor wound healing, and those in whom surgery would be deforming or destroy function (e.g., cancers of the face and fingers). Moreover, intralesional IFN can be used to shrink tumors prior to surgery or for the treatment of positive margins after surgical excision [1].

Although IFN-*β* has shown more potent anti-proliferative, proapoptotic, and immunomodulatory effects than IFN-*α* in many of the above studies, further clinical trials involving larger numbers of patients are needed to establish the therapeutic profile of IFN-*β* [48, 74]. Moreover, the finding that melanoma cell lines differ in their sensitivity to the same IFN may explain variations in clinical response. High-dose interferon therapy produces a clinical response and achieves relapse-free survival in only 20–33% of patients with operable high risk or metastatic melanoma [72], and therefore a better understanding of its antitumor mechanism of action would enable more selective application of this therapy to those patients who are most likely to benefit.

## Figures and Tables

**Table 1:** Effect of type I Interferon on normal keratinocytes and melanocytes.

Type of effect	Description of effect	References
Antiproliferative	IFNs-*α*, *β* inhibit the growth of human keratinocytes *in vitro* and promote keratinocyte terminal differentiation. IFNs-*α*, *β* inhibit the growth of human melanocytes *in vitro*, with IFN-*β* having a greater effect than IFN-*α*.	[17–20]

Antiangiogenesis	IFN sensitivity of surrounding tissue is necessary for inhibition of angiogenesis around gelfoam sponges implanted in mice.	[21, 22]

Immunomodulatory	IFN-*α* upregulates class I, but not class II, MHC antigen expression in cultured human keratinocytes. IFNs-*α*, *β* induce increased expression of class I MHC antigens in cultured human melanocytes, with IFN-*β* having a greater effect than IFN-*α*.	[20, 23]

**Table 2:** Effect of type I Interferon on cutaneous squamous cell carcinoma.

Type of effect	Description of effect	References
Antiproliferative	IFN-*β* has greater antiproliferative effect than IFN-*α* in SCL-1 cells. IFN-*α* induced a partial G1/0 arrest in SRB12-p9 cells. SCC-12B.2 cell line is less sensitive than normal keratinocytes to growth inhibitory effects of IFNs-*α*, *β*	[17, 24–26]

Proapoptotic	IFN-*α* led to a twofold increase in apoptosis in SRB12-p9 cells compared to controls. Upon IFN-*α* treatment, SRB-12 cells exhibited ultrastructural evidence of apoptosis on microscopy.	[26, 27]

Immunomodulatory	IFNs-*α*, *β* reduced IFN-*γ*-induced HLA-DR expression in A431 cells.	[25]

Miscellaneous	Compared to normal skin, there was decreased staining intensity for ISGF3 proteins in not only specimens of human skin SCCs but also specimens of actinic keratoses. Cell growth inhibitory effect of IFN-*α* requires an intact STAT2 protein.	[4, 5, 28]

**Table 3:** Effect of type I interferon on basal cell carcinoma.

Type of effect	Description of effect	References
Antiproliferative	IFN-*α* upregulates opioid growth factor receptor expression in BCC cell lines.	[29, 30]

Proapoptotic	IFN-*α* leads to coexpression of CD95L and CD95 in nodular BCCs, leading to cell death by suicide and fratricide.	[31]

Immunomodulatory	IFN-*α* leads to decreased mRNA levels of the immunosuppressive cytokine IL-10 in BCCs. Treatment with IFN-*α* led to increased proportion of CD3^+^ cells within peri- and intratumoral infiltrates of BCCs as well as an increase in the CD4/CD8 ratio in both peri- and intratumoral infiltrates.	[32–35]

**Table 4:** Effect of type I interferon on melanoma.

Type of effect	Description of effect	References
Antiproliferative	IFN-*β* has greater antiproliferative effect than IFN-*α* in the cell lines SK-MEL-1, 2, 3, 24, and 28; LiBr; Hs294T; HT144; and JKM86-4, 5, 8, and 9.	[20, 36–46]

Proapoptotic	IFN-*β* induced apoptosis dose-dependently in multiple cell lines, with a greater effect than IFN-*α* at all time points. In WM9 cells, IFN-*β* led to increased levels of cyt c and increased activity of caspases 3, 8, and 9. IFN-*β* induces TRAIL mRNA expression and XAF-1, which is a negative regulator of XIAP.	[37, 39, 42, 44, 47]

Anti-angiogenesis	IFN-*β* increases serum levels of antiangiogenic cytokines and decreases serum levels of pro-angiogenic cytokines. IFNs-*α*, *β* decrease intracellular and secretory levels of VEGF in multiple cell lines, with a superior effect by IFN-*α*. In human melanoma xenograft tumors, IFNs-*α*, *β* similarly decrease microvessel density but IFN-*α* has a superior effect over IFN-*β* in decreasing lymphatic vessel density.	[37, 39, 48, 49]

Immunomodulatory	*IFN-α* *:* tumor infiltration with CD4^+^ cells is not only required for the therapeutic effect of IFN-*α* but is also a consequence of treatment with IFN-*α*. Use of IFN-*α* as an adjuvant in the vaccination of metastatic melanoma patients has resulted in disease stabilization. Responders to IFN-*α* therapy show restoration of immune responses to almost normal levels based on *in vitro* studies of T cell function and cytokine production. Metastases from IFN-*α*-treated patients show a low TNF-*α* staining score compared to those from untreated patients. IFN-*α* is a potent stimulator of antimelanoma lytic activity via natural killer cells and cytotoxic T lymphocytes. In melanoma cell lines, IFN-*α* significantly enhances class I, but not class II, MHC expression. IFN-DCs may be promising adjuvants for cancer immunotherapy targeting melanoma. *IFN-β:* treatment with IFN-*β* leads to increased T cell infiltration of interstitium and tumor nests. IFN-*β* has a greater effect than IFN-*α* in inhibiting the TNF-*α*-mediated upregulation of IL-8, a growth factor for melanoma cells. IFN-*β* augments NK cell-mediated cytotoxicity against melanoma cell lines. In melanoma cell lines, IFN-*β* significantly enhances class I, but not class II, MHC expression, and augments expression of tumor-associated antigens.	[43, 50–58, 60–66, 68]

Miscellaneous	Proinflammatory cytokines promote degradation of IFNAR1, leading to decreased tumor responsiveness to IFN. IFN-*α* treatment leads to decreased detection of Stat3 homo- and heterodimers in atypical nevi as well as dephosphorylation of Stat3 protein in atypical nevi. Higher pretreatment pSTAT1/pSTAT3 ratios in tumor cells were associated with longer overall survival in stage IIIB patients. IFN signaling patterns in peripheral blood lymphocytes, as measured by STAT1 activation, can be used to select patients for high dose interferon therapy. IFN-*β* maintenance therapy was shown to significantly increase overall and relapse-free survival in a clinical study of stage II-III melanoma patients.	[69–73]
